# Evolution of a Non-Hermitian Quantum Single-Molecule Junction at Constant Temperature

**DOI:** 10.3390/e23020147

**Published:** 2021-01-25

**Authors:** Andrea Grimaldi, Alessandro Sergi, Antonino Messina

**Affiliations:** 1 Dipartimento di Scienze Matematiche e Informatiche, Scienze Fisiche e Scienze della Terra, Università degli Studi di Messina, 98166 Messina, Italy; angrimaldi@unime.it; 2 Istituto Nazionale di Fisica Nucleare, Sez. di Catania, 95123 Catania, Italy; 3 Institute of Systems Science, Durban University of Technology, Durban 4000, South Africa; 4 Dipartimento di Matematica ed Informatica dell’Università di Palermo, 90123 Palermo, Italy; antonino.messina@unipa.it

**Keywords:** molecular junction, non-Hermitian quantum mechanics, open quantum system dynamics, quantum thermodynamics, 31.15.xg, 02.60.Cb, 05.30.-d, 05.60.Gg, 03.67.Pp, 80M99, 81-08, 81-10, 81P99

## Abstract

This work concerns the theoretical description of the quantum dynamics of molecular junctions with thermal fluctuations and probability losses. To this end, we propose a theory for describing non-Hermitian quantum systems embedded in constant-temperature environments. Along the lines discussed in [A. Sergi et al., Symmetry 10 518 (2018)], we adopt the operator-valued Wigner formulation of quantum mechanics (wherein the density matrix depends on the points of the Wigner phase space associated to the system) and derive a non-linear equation of motion. Moreover, we introduce a model for a non-Hermitian quantum single-molecule junction (nHQSMJ). In this model the leads are mapped to a tunneling two-level system, which is in turn coupled to a harmonic mode (i.e., the molecule). A decay operator acting on the two-level system describes phenomenologically probability losses. Finally, the temperature of the molecule is controlled by means of a Nosé-Hoover chain thermostat. A numerical study of the quantum dynamics of this toy model at different temperatures is reported. We find that the combined action of probability losses and thermal fluctuations assists quantum transport through the molecular junction. The possibility that the formalism here presented can be extended to treat both more quantum states (∼10) and many more classical modes or atomic particles ($∼10^{3}-10^{5}$) is highlighted.

## 1. Introduction

Molecular junctions are nano-devices composed of metal or semiconductor electrodes known as leads [1,2]. A single molecule simulates a conducting bridge between the leads. Such a nanostructure is almost perfectly suited to study non-equilibrium quantum transport [1,2].

Various approaches satisfactorily befit the investigation of the phenomenology of molecular junctions [2]. In this work, we introduce a simple toy model of a molecular junction [2] in order to perform a qualitative study aimed at singling out universal features of such systems. To this end, the operator-valued Wigner formulation of quantum mechanics [3,4,5] is particularly useful to embed quantum toy-models in quantum phase space baths since it can simultaneously describe dissipative phenomena and thermal fluctuations. A quantum-classical molecular junction model has been studied in [6].

In general, theories of quantum transport offer an acceptable representation of excitations and state population transfer. They usually assume that what is transferred does not disintegrate or disappear from the system. Such a physical property is expressed through the conservation of probability. On the contrary, by definition, the probability in quantum systems with gain and loss is not conserved. Such a feature can be incorporated in the theory by introducing non-Hermitian Hamiltonians [7,8,9,10,11,12]. Recently, the development of non-Hermitian quantum mechanics (NHQM) has been propelled by various experiments on many systems (see [13,14] and references therein). While in this work a non-equilibrium quantum statistical mechanics of molecular junctions is adopted, linear equations of motion are used in [10,11,12]. Conceptually, this can be understood in terms of the different theoretical targets: In [10,11,12] the target is to study non-equilibrium transport. Instead, our work is aimed at showing the possibility of a statistical mechanical description for non-Hermitian quantum systems in classical bath [15].

Since probability non-conserving quantum systems are open quantum systems [14], it is natural to formulate NHQM in terms of the density matrix [15,16,17,18,19,20,21,22]. Typically, a non-Hermitian Hamiltonian is either derived by means of the Feshbach formalism [23,24] or it is postulated as an ansatz (see [15,16,17,18,19,20,21,22]). It is remarkable that there is a third possibility according to which a non-Hermitian Hamiltonian may arise from stroboscopic measurements performed on an ancilla-subsystem added to a quantum system, a very interesting process capable of generating entanglement [25]. Given a non-Hermitian Hamiltonian and assuming that the Schrödinger equation is still valid, one derives a probability non-conserving equation of motion for the density matrix of the system [25]. However, in order to meet the need of establishing a proper quantum statistical theory, this non-Hermitian equation of motion for the density matrix is generalized into a non linear-one [15,16,17,18,19,20,21,22]. Essentially, this is the origin of the difference between the linear approach of [10,11,12] and the non-linear approach of [15,16,17,18,19,20,21,22].

Various investigations on thermal effects in molecular junctions are found in the literature [26,27,28]. However, to the best of our knowledge, thermal effects have been investigated separately from probability losses of molecular junctions. In light of the previous considerations, the purpose of this work is to combine the non-linear equation for the density matrix of a system with a non-Hermitian Hamiltonian and temperature control in quantum phase space dynamics [29]. Temperature control is technically implemented by means of the Nosé-Hoover chain [30] thermostat, formulated in terms of quasi-Lie brackets [3]. The unified formalism here reported is developed for studying a greater variety of phenomena than those accessible to theories treating thermal fluctuations and probability losses separately. In detail, the theory is obtained through the density matrix approach to non-Hermitian quantum mechanics [15,16,17,18,19,20,21,22] and it is expressed in terms of the operator-valued formulation of quantum mechanics [3,4,5,15]. For clarity, we highlight the three mathematical ingredients that must be combined for building our formulation. First, we need an approach for describing quantum operators in terms of operator-valued Wigner phase space functions. The conceptual framework of this specific theory assumes an Eulerian picture where a Hilbert space, or a space of quantum discrete states, is defined for each point X=(Q,P) of phase space. Accordingly, the operator-valued Wigner function [3,4,5] may also be called phase-space-dependent density matrix. The consistent coupling of quantum and phase space degrees of freedom requires that quantum dynamics in the Hilbert space associated to *X* also depends non-locally on the quantum dynamics associated to different points $X^{\prime }$. Likewise, the dynamics of *X* does not only depend on its quantum space but it also depends on the quantum spaces of other points $X^{\prime }$ and vice versa. In practice, such a dependence can be implemented through a variety of algorithms [31,32,33,34]. When the Hamiltonian is quadratic, one can expand the operator-valued Moyal bracket [35,36] up to linear order in *ℏ* and obtain quantum equations of motion [37].

The second ingredient entering our theory is the requirement that the degrees of freedom in phase space evolve in time satisfying the constraint determined by the constant thermodynamic temperature. The temperature control of the classical degrees of freedom is achieved in silico by means of the Nosé-Hoover chain algorithm [30]. We find such an approach advantageous because the formulation of the Nosé-Hoover chain algorithm can be realized within the theoretical framework given by the quasi-Lie brackets [3]. Such brackets allow one to generalize the Nosé-Hoover chain algorithm, originally formulated for classical systems only, to the more general case of the operator-valued formulation of quantum mechanics [29,35,36].

The third ingredient, of course, is that the modeling of the leads provides a non-Hermitian quantum system.

When all these three theoretical features are simultaneously taken into account, we obtain an equation that generalizes the one arising from the quantum-classical quasi-Lie bracket [3] because it can describe probability gain/loss [15] too. Moreover, since such an equation keeps the thermodynamic temperature of the phase space degrees of freedom constant by means of the Nosé-Hoover chain algorithm [3,30,35,36], it generalizes the equations used in [15,16,17,18,19,20,21,22], which did not implement any thermodynamic constraint.

In this paper we investigate the time evolution of a single-molecule junction toy-model represented by a non-Hermitian Hamiltonian with a parametric phase space dependence. The isolated leads are represented by a two-level system. In this situation, the transport of the occupation probability of one or the other state can only occur through quantum tunneling. The molecule connecting the two leads is modeled by a harmonic oscillator. When we consider only the coupling between the harmonic mode and the two-level system, the total system is considered closed. In this case, transport can take place both via tunneling and via the channel given by the oscillator. In order to build a complete non-Hermitian quantum single-molecule junction model at constant temperature, the closed spin-boson system is transformed into an open quantum system by means of two theoretical steps. The first step is to consider a decay operator acting on the two-level system. This operator introduces another transport channel that does not conserve probability and then breaks time-reversibility.

The second step is to put the harmonic mode into contact with a heat bath. Of course, this condition forces the molecule to have the same temperature of the bath and to experience fluctuations according to the canonical ensemble probability distribution. In practice, the action of the constant-temperature bath on the molecule is implemented by means of the Nosé-Hoover chain thermostat. For this reason, in the following we will use wording such as “heat bath”, “constant-temperature environment”, or “Nosé-Hoover chain thermostat” interchangeably.

The final complete non-Hermitian Hamiltonian is designed to represent two lossy leads interacting with a thermalized molecule.

The organization of the paper is the following: In Section 2 we give a short summary of the density matrix approach to the dynamics of a system governed by a non-Hermitian Hamiltonian. In Section 3 we describe briefly how to treat non-Hermitian quantum systems interacting with classical degrees of freedom. We present the main theoretical results of this work in Section 4, where we introduce the mathematical formalism describing the quantum dynamics of a non-Hermitian quantum system interacting with classical degrees of freedom at constant temperature. In Section 5 we introduce the non-Hermitian quantum single-molecule junction model. The results of the numerical calculations are reported in Section 6. Finally, our conclusions are given in Section 7.

## 2. Quantum Dynamics of Non-Hermitian Systems

Consider a quantum system described by a non-Hermitian Hamiltonian operator:

(1) $$\hat{H}=\hat{H}-i\hat{\Gamma },$$

where $\hat{H}$ and $\hat{\Gamma }$ are Hermitian operators ($\hat{\Gamma }$ is the decay operator). In the presence of a probability sink or source, the dynamics of the system is defined in terms of the following equations (we set ℏ=1):

(2) $$\begin{array}{ccc} \frac{d}{dt}|\Psi \left(t\right)\rangle & = & -i\hat{H}|\Psi \left(t\right)\rangle \;, \end{array}$$

(3) $$\begin{array}{ccc} \frac{d}{dt}\langle \Psi \left(t\right)| & = & i\langle \Psi \left(t\right)|{\hat{H}}^{†}\;, \end{array}$$

where ∣Ψ(t)〉 and 〈Ψ(t)∣ are state vectors of the system. Equations (2) and (3) reduce to the standard Schrödinger equation when $\hat{\Gamma }=0$.

The density matrix approach to non-Hermitian quantum systems [15,16,17,18,19,20,21,22] is obtained upon introducing at any *t* > 0 a Hermitian, semipositive defined, but non-normalized density matrix:

(4) $$\hat{\Omega }\left(t\right)=|\Psi \left(t\right)\rangle \langle \Psi \left(t\right)|\;.$$

The equation of motion for $\hat{\Omega }$ [16,20,22] is obtained by taking the time derivative of Equation (4) and using Equation (1):

(5) $$\frac{d}{dt}\hat{\Omega }\left(t\right)=-i\left[\hat{H},\hat{\Omega }\left(t\right)\right]-{\left[\hat{\Gamma },\hat{\Omega }\left(t\right)\right]}_{+}\;,$$

where […,…] is the commutator and ${\left[\ldots ,\ldots \right]}_{+}$ is the anticommutator. Equation (5) is linear but, nevertheless, it describes an open quantum system. This is possible since it is defined in terms of a decay operator that physically describes the presence of probability sources/sinks, arising from the hidden system with which the open quantum system interacts.

Equation (5) is non-Hermitian, breaks time reversibility and does not conserve the trace of $\hat{\Omega }\left(t\right)$. In fact, from Equation (5) one can easily derive:

(6) $$\frac{d}{dt}\mathrm{Tr}\left(\hat{\Omega }\left(t\right)\right)=-2\mathrm{Tr}\left(\hat{\Gamma }\hat{\Omega }\left(t\right)\right)\;.$$

In order to obtain a well-founded statistical mechanics of non-Hermitian quantum systems, one can introduce the normalized density matrix:

(7) $$\hat{\rho }\left(t\right)=\frac{\hat{\Omega }\left(t\right)}{\mathrm{Tr}\left(\hat{\Omega }\left(t\right)\right)}\;.$$

This operator $\hat{\rho }\left(t\right)$ obeys the non-linear equation:

(8) $$\frac{d}{dt}\hat{\rho }\left(t\right)=-i\left[\hat{H},\hat{\rho }\left(t\right)\right]-{\left[\hat{\Gamma },\hat{\rho }\left(t\right)\right]}_{+}+2\hat{\rho }\left(t\right)\mathrm{Tr}\left(\hat{\Gamma }\hat{\rho }\left(t\right)\right)\;.$$

As a consequence of its very definition and of Equation (8), $\mathrm{Tr}(\hat{\rho }\left(t\right))=1$ at all times. Equation (8) breaks time reversal symmetry and is non-linear. In practice, the formalism trades off the non-conservation of probability in Equation (5) with the non-linearity of Equation (8). As the linear Equation (5), the non-linear Equation (8) describes the dynamics of an open quantum system. Consistently, averages of arbitrary operators, e.g., $\hat{\chi }$, can be calculated by means of the normalized density matrix $\hat{\rho }$ as:

(9) $${\langle \hat{\chi }\rangle }_{\left(t\right)}=\mathrm{Tr}\left(\hat{\rho }\left(t\right)\hat{\chi }\right)\;.$$

Equation (9) establishes the foundations of the statistical interpretation of non-Hermitian quantum mechanics.

## 3. Non-Hermitian Quantum Systems Set in Phase Space

Non-Hermitian quantum systems can be embedded in phase space [15]. In the following, we sketch the derivation given in [15]. Let us consider a system described by a hybrid set of coordinates $\left(\hat{x},X\right)$, where $\hat{x}$ are quantum coordinates (operators) and X=(Q,P) are phase space coordinates (an obvious multidimensional notation is used in order not to clutter formulas with too many indices). In this specific case, the *X*s describe the phase space coordinates of the system. As we have already discussed in the Introduction, the description of the system, using the operator-valued-formulation of quantum mechanics, can be established via an Eulerian point of view according to which a space of state vectors is defined for any point *X* of phase space [3,4,5] so that $\hat{x}$ (and operators defined in terms of $\hat{x}$) can act on it. In such a hybrid space there are operators that depend only on $\hat{x}$, which we denote with the symbol x^ on top of the operator, e.g., $\hat{\Gamma }$. There are pure phase space functions which we denote by their arguments, e.g., $H_{B}\left(X\right)$, or G(X,t) when there is also an explicit time dependence. Finally, there are operators that depend both on $\hat{x}$ and (parametrically) on *X*. We denote the latters with the symbol x˜ on top, e.g., $\tilde{\Omega }\left(t\right)\equiv \hat{\Omega }\left(X,t\right)$.

In the non-Hermitian case, we consider a Hamiltonian operator with parametric dependence on phase space:

(10) $$\begin{array}{ccc} \tilde{H} & = & \hat{H}+H_{B}\left(X\right)+{\tilde{H}}_{I}-i\hat{\Gamma }\\ & = & {\tilde{H}}_{S}-i\hat{\Gamma }\;, \end{array}$$

where $\hat{H}$ is the Hermitian Hamiltonian of the quantum subsystem, $H_{B}\left(X\right)$ is the Hamiltonian of the degrees of freedom in phase space, ${\tilde{H}}_{I}$ describes the interaction between the quantum system and the bath, and ${\tilde{H}}_{S}=\hat{H}+H_{B}\left(X\right)+{\tilde{H}}_{I}$. Of course, $\tilde{H}$ is the total non-Hermitian Hamiltonian of the system with $\hat{\Gamma }$ as the decay operator.

When the operator $\hat{\Gamma }$ acts on the quantum dynamical variables of the non-Hermitian quantum system, it has been shown [15] that, as a generalization of the equations given in [3,4,5], the equation of motion becomes:

(11) $$\frac{\partial }{\partial t}\tilde{\Omega }\left(t\right)=-i\left[{\tilde{H}}_{S},\tilde{\Omega }\left(t\right)\right]-{\left[\hat{\Gamma },\tilde{\Omega }\left(t\right)\right]}_{+}+\frac{1}{2}{\left\{{\tilde{H}}_{S},\tilde{\Omega }\left(t\right)\right\}}_{B}-\frac{1}{2}{\left\{\tilde{\Omega }\left(t\right),{\tilde{H}}_{S}\right\}}_{B}\;,$$

where $\tilde{\Omega }\left(t\right)$ is the phase-space-dependent non-normalized density matrix of the system and

(12) $$B=\left[\begin{array}{cc} 0 & 1\\ -1 & 0 \end{array}\right]$$

is the symplectic matrix written in block form. Using it, one can write the Poisson bracket in the form adopted in Equation (18):

(13) $${\left\{{\tilde{H}}_{S},\tilde{\Omega }\left(t\right)\right\}}_{B}\equiv \sum_{I,J=1}^{2N}\frac{\partial {\tilde{H}}_{S}}{\partial X_{I}}B_{IJ}\frac{\partial \tilde{\Omega }\left(t\right)}{\partial X_{J}}\;.$$

The dimension of phase space is 2N.

The trace of the phase-space-dependent density matrix $\tilde{T}r$ is defined as:

(14) $$\tilde{T}r\left(\tilde{\Omega }\left(t\right)\right)={\mathrm{Tr}}^{\prime }\int dX\tilde{\Omega }\left(X,t\right)\;,$$

where ${\mathrm{Tr}}^{\prime }$ denotes a partial trace over the quantum dynamical variables $\hat{x}$ and ∫dX is the integral in phase space. The trace $\tilde{T}r\left(\tilde{\Omega }\left(t\right)\right)$ obeys the equation of motion:

(15) $$\frac{d}{dt}\tilde{T}r\left(\tilde{\Omega }\left(t\right)\right)=-2\tilde{T}r\left(\hat{\Gamma }\tilde{\Omega }\left(t\right)\right)\;.$$

The result given in Equation (15) is proven in Appendix A.

Let us now introduce a phase-space-dependent normalized density matrix $\tilde{\rho }\left(X,t\right)$:

(16) $$\tilde{\rho }\left(X,t\right)=\frac{\tilde{\Omega }\left(X,t\right)}{\tilde{T}r\left(\tilde{\Omega }\left(X,t\right)\right)}\;.$$

Quantum statistical averages of an arbitrary phase-space-dependent operator $\tilde{\chi }$ can now be calculated as:

(17) $${\langle \tilde{\chi }\rangle }_{\left(t\right)}=\tilde{T}r\left(\tilde{\rho }\left(t\right)\tilde{\chi }\right)\;.$$

The phase-space-dependent normalized density matrix obeys the non-linear equation of the motion below:

(18) $$\frac{\partial }{\partial t}\tilde{\rho }\left(t\right)=-i\left[{\tilde{H}}_{S},\tilde{\rho }\left(t\right)\right]-{\left[\hat{\Gamma },\tilde{\rho }\left(t\right)\right]}_{+}+2\hat{\rho }\left(t\right)\tilde{T}r\left(\hat{\Gamma }\tilde{\rho }\left(t\right)\right)+\frac{1}{2}{\left\{{\tilde{H}}_{S},\tilde{\rho }\left(t\right)\right\}}_{B}-\frac{1}{2}{\left\{\tilde{\rho }\left(t\right),{\tilde{H}}_{S}\right\}}_{B}\;.$$

## 4. Non-Hermitian Quantum Systems Set in a Constant-Temperature Phase Space

Equations (11) and (18) describe the dynamics of non-Hermitian quantum systems embedded in phase space [15]. When considering open quantum systems in more general settings, it is common to consider the effects of thermal fluctuations. Phase space coordinates undergo thermal fluctuations when their microscopic equilibrium state is described by the canonical distribution function. In this ensemble the thermodynamic temperature is constant. Within the framework of the operator-valued Wigner formulation of quantum mechanics [3,4,5], temperature constraints are efficiently formulated by means of quasi-Lie brackets [3,35,36]. In the classical case, deterministic thermostats, such as the Nosé-Hoover chain thermostat [30], are also implemented by means of quasi-Lie brackets [38,39,40]. Below, we briefly explain how this is achieved. In silico (i.e., on the computer), temperature control is realized by augmenting the dimensions of phase space using just a few additional degrees of freedom. The new higher dimensional phase space is known as extended phase space. We indicate points in the extended phase space by means of the symbol $X^{e}$. In the case of the Nosé-Hoover chain thermostat, upon introducing two fictitious coordinates $\Lambda_{1},\Lambda_{2}$ with their associated momenta $\Pi_{1},\Pi_{2}$, we have the following point in the extended phase space:

(19) $$X^{e}=\left(Q,\Lambda_{1},\Lambda_{2},P,\Pi_{1},\Pi_{2}\right)\;.$$

To lighten the notation, the phase space coordinates of the Nosé-Hoover chain thermostat are denoted as $Y=(\Lambda_{1},\Lambda_{2},\Pi_{1},\Pi_{2})$. For the reader, the symbol Y denotes a calligraphic *Y*. A generic operator acting upon the extended phase space is identifiable by the apex *e*. Considering a classical system with a potential term V(Q) and Hamiltonian $H_{B}\left(X\right)=P^{2}/2+V\left(Q\right)$, the extended phase space Hamiltonian (see [38,39,40] and references therein) includes the Nosé-Hoover chain energy [30,38] and is defined as:

(20) $$\begin{array}{ccc} H^{e}\left(X^{e}\right) & = & H_{B}\left(X\right)+\sum_{K=1}^{2}\left(\frac{\Pi_{K}^{2}}{2\mu_{K}}\right)+gk_{B}T\Lambda_{1}+k_{B}T\Lambda_{2}\\ & = & H_{B}\left(X\right)+H_{nhc}\left(Y\right)\;, \end{array}$$

where $\mu_{K}$ are fictitious inertial parameters, *T* is the termodynamic temperature, $k_{B}$ is the Boltzmann constant, and *N* is the number of physical coordinates *Q* that are thermalized. The equation of motion in extended phase space can be written as:

(21) $${\dot{X}}_{K}^{e}={\left\{X_{K}^{e},H^{e}\right\}}_{B^{e}}=\sum_{I,J=1}^{2(N+2)}\frac{\partial X_{K}^{e}}{\partial X_{I}^{e}}B_{IJ}^{e}\frac{\partial H^{e}}{\partial X_{J}^{e}}=\sum_{J=1}^{2(N+2)}B_{KJ}^{e}\frac{\partial H^{e}}{\partial X_{J}^{e}}\;,$$

where the first and second equality define a quasi-Lie bracket. The last equality enlighten the matrix structure of the equations of motion. This last form also allows one to compactly define the compressibility of phase space:

(22) $$\kappa^{e}\equiv \sum_{I=1}^{2(N+2)}\frac{\partial {\dot{X}}_{I}^{e}}{\partial X_{I}^{e}}=\sum_{I,J=1}^{2(N+2)}\frac{\partial B_{IJ}^{e}}{\partial X_{I}^{e}}\frac{\partial H^{e}}{\partial X_{J}^{e}}=-N\frac{{\dot{\Pi }}_{1}}{\mu_{1}}-\frac{{\dot{\Pi }}_{2}}{\mu_{2}}\;$$

The antisymmetric matrix $B^{e}$, entering Equations (21) and (22), is defined as:

(23) $$B^{e}=\left[\begin{array}{cccccc} 0 & 0 & 0 & 1 & 0 & 0\\ 0 & 0 & 0 & 0 & 1 & 0\\ 0 & 0 & 0 & 0 & 0 & 1\\ -1 & 0 & 0 & 0 & -P & 0\\ 0 & -1 & 0 & P & 0 & -\Pi_{1}\\ 0 & 0 & -1 & 0 & \Pi_{1} & 0 \end{array}\right],$$

is an antisymmetric phase-space-dependent matrix generalizing the symplectic matrix in Equation (12). Equation (21) is called a quasi-Hamiltonian equation because, while conserving $H^{e}\left(X^{e}\right)$, it cannot be derived from the Hamiltonian formalism alone: In order to write the equation of motion (21), together with $H^{e}\left(X^{e}\right)$, one also needs the antisymmetric matrix $B^{e}$ in Equation (23). The equation of motion for the distribution function $f(X^{e},t)$ is:

(24) $$\frac{\partial }{\partial t}f\left(t\right)=-{\left\{f\left(t\right),H^{e}\right\}}_{B^{e}}-\kappa^{e}f\left(t\right)\;.$$

Given the discussion above, our task becomes that of generalizing Equation (24) to a non-Hermitian quantum system. The phase-space-dependent Hamiltonian of the quantum system must be defined on the extended phase space of the Nosé-Hoover chain thermostat:

(25) $$\begin{array}{ccc} {\tilde{H}}^{e} & = & \hat{H}+H_{B}\left(X\right)+{\tilde{H}}_{I}+H_{\mathrm{nhc}}\left(Y\right)-i\hat{\Gamma }\\ & = & {\tilde{H}}_{S}^{e}-i\hat{\Gamma }\;. \end{array}$$

A phase-space-dependent non-normalized density matrix ${\tilde{\Omega }}^{e}\left(t\right)$ can be introduced as well. We postulate that ${\tilde{\Omega }}^{e}\left(t\right)$ obeys the equation of motion:

(26) $$\frac{\partial }{\partial t}{\tilde{\Omega }}^{e}\left(t\right)=-i\left[{\tilde{H}}_{S}^{e},{\tilde{\Omega }}^{e}\left(t\right)\right]-{\left[\hat{\Gamma },{\tilde{\Omega }}^{e}\left(t\right)\right]}_{+}+\frac{1}{2}{\left\{{\tilde{H}}_{S}^{e},{\tilde{\Omega }}^{e}\right\}}_{B^{e}}-\frac{1}{2}{\left\{{\tilde{\Omega }}^{e},{\tilde{H}}_{S}^{e}\right\}}_{B^{e}}-\kappa_{S}^{e}{\tilde{\Omega }}^{e}\left(t\right)\;.$$

In the absence of the quantum degrees of freedom, Equation (26) reduces correctly to Equation (24). Moreover, when $(\Lambda_{I},\Pi_{I})\to 0$, with I=1,2, (which means that the Nosé-Hoover thermostat is not applied), Equation (26) reduces unerringly to Equation (11).

The trace $\tilde{T}r^{e}$ of the density matrix ${\tilde{\Omega }}^{e}\left(t\right)$ involves an integral over the extended phase space:

(27) $$\tilde{T}r^{e}\left({\tilde{\Omega }}^{e}\left(t\right)\right)={\mathrm{Tr}}^{\prime }\int dX^{e}{\tilde{\Omega }}^{e}\left(X^{e},t\right)\;.$$

The equation of motion of the trace defined in Equation (27) has the same structure of Equation (15):

(28) $$\frac{d}{dt}\tilde{T}r^{e}\left({\tilde{\Omega }}^{e}\left(t\right)\right)=-2\tilde{T}r^{e}\left(\hat{\Gamma }{\tilde{\Omega }}^{e}\left(t\right)\right)\;.$$

Appendix B shows how Equation (28) is obtained.

The phase-space-dependent normalized density matrix in the extended phase space, ${\tilde{\rho }}^{e}\left(X^{e},t\right)$, is defined in analogy with Equation (16) as:

(29) $${\tilde{\rho }}^{e}\left(X^{e},t\right)=\frac{{\tilde{\Omega }}^{e}\left(X^{e},t\right)}{\tilde{T}r^{e}\left({\tilde{\Omega }}^{e}\left(X^{e},t\right)\right)}\;.$$

In analogy with Equation (17), quantum statistical averages are calculated as:

(30) $${\langle {\tilde{\chi }}^{e}\rangle }_{\left(t\right)}^{e}=\tilde{T}r^{e}\left({\tilde{\rho }}^{e}\left(t\right){\tilde{\chi }}^{e}\right)\;.$$

The phase-space-dependent normalized density matrix in Equation (29) obeys the non-linear equation of motion:

(31) $$\begin{array}{ccc} \frac{\partial }{\partial t}{\tilde{\rho }}^{e}\left(t\right) & = & -i\left[{\tilde{H}}_{S}^{e},{\tilde{\rho }}^{e}\left(t\right)\right]-{\left[\hat{\Gamma },{\tilde{\rho }}^{e}\left(t\right)\right]}_{+}+2{\tilde{\rho }}^{e}\left(t\right)\tilde{T}r^{e}\left(\hat{\Gamma }{\tilde{\rho }}^{e}\left(t\right)\right)\\ & + & \frac{1}{2}{\left\{{\tilde{H}}_{S}^{e},{\tilde{\rho }}^{e}\right\}}_{B^{e}}-\frac{1}{2}{\left\{{\tilde{\rho }}^{e},{\tilde{H}}_{S}^{e}\right\}}_{B^{e}}-\kappa_{S}^{e}{\tilde{\rho }}^{e}\left(t\right)\;. \end{array}$$

We remark that Equations (26) and (31) are two important results of this work since these equations define the dynamics of a non-Hermitian quantum system embedded in a classical bath at constant temperature.

## 5. Model of a Non-Hermitian Quantum Single-Molecule Junction at Constant Temperature

In this section we formulate a molecular junction toy-model described by a non-Hermitian Hamiltonian denoted by ${\tilde{H}}^{m}$. The various energy contributions entering the Hermitian terms in part of the Hamiltonian model are:

(32) $$\begin{array}{ccc} {\hat{H}}^{m} & = & -\Delta {\hat{\sigma }}_{z}\;, \end{array}$$

(33) $$\begin{array}{ccc} H_{B}^{m}\left(X\right) & = & \frac{P^{2}}{2}+\frac{\omega^{2}}{2}Q^{2}\;, \end{array}$$

(34) $$\begin{array}{ccc} {\tilde{H}}_{I}^{m}\left(Q\right) & = & -cQ{\hat{\sigma }}_{x}\;, \end{array}$$

(35) $$\begin{array}{ccc} H_{\mathrm{nhc}}^{m}\left(Y\right) & = & \sum_{K=1}^{2}\left(\frac{\Pi_{K}^{2}}{2\mu_{K}}+k_{B}T\Lambda_{K}\right)\;. \end{array}$$

The two-level system Hamiltonian ${\hat{H}}^{m}$ describes quantum transport between the leads in terms of the variation of the population of a ground and an excited state. The extended phase space point $X^{m}$ is defined as in Equation (21) but the model considers a single *Q* coordinate and its conjugate momentum Π. Transport in the isolated two-level system takes place through tunneling. The inclusion in the model description of the decay operator ${\hat{\Gamma }}^{m}$ opens a channel through which the population of the states can disappear forever from the two-level system. The explicit form of ${\hat{\Gamma }}^{m}$ will be given later on when we transform to the adiabatic basis. This subsystem is also coupled to a harmonic mode, with free Hamiltonian $H_{B}^{m}\left(Q\right)$, by means of an interaction Hamiltonian ${\tilde{H}}_{I}^{m}$. The harmonic mode opens another channel for the transport of the population between the leads. In addition to this, the harmonic mode is embedded in a thermal bath via the Nosé-Hoover chain thermostat with energy $H_{\mathrm{nhc}}^{m}$. The complete non-Hermitian Hamiltonian model ${\tilde{H}}^{m}$ reads:

(36) $$\begin{array}{ccc} {\tilde{H}}^{m}\left(X^{m}\right) & = & {\hat{H}}^{m}+H_{B}^{m}\left(X\right)+{\tilde{H}}_{I}^{m}\left(Q\right)+H_{\mathrm{nhc}}^{m}\left(Y\right)-i{\hat{\Gamma }}^{m}\\ & = & {\tilde{H}}_{S}^{m}\left(X^{m}\right)-i{\hat{\Gamma }}^{m}\;, \end{array}$$

where the last equality also defines the Hermitian part of the Hamiltonian model, i.e.,

(37) $$\begin{array}{c} {\tilde{H}}_{S}^{m}\left(X^{m}\right)={\hat{H}}^{m}+H_{B}^{m}\left(X\right)+{\tilde{H}}_{I}\left(Q\right)+H_{\mathrm{nhc}}^{m}\left(Y\right)\;. \end{array}$$

**Figure 1:**
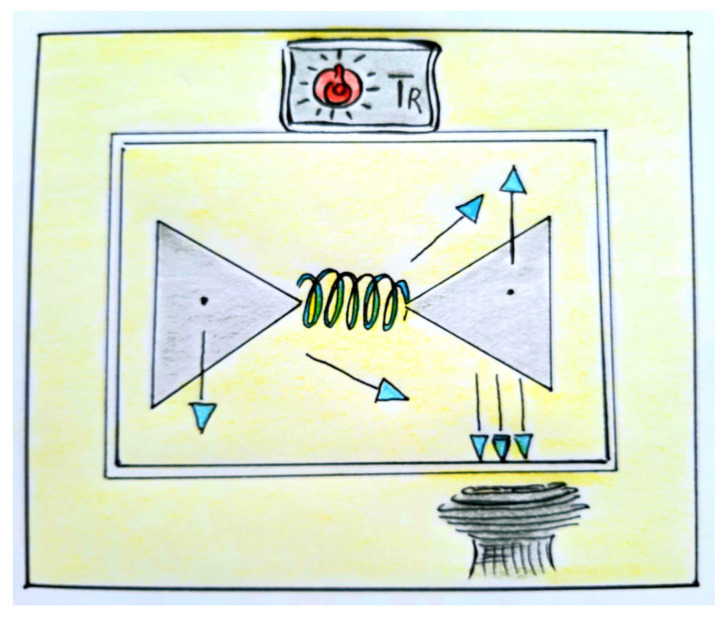
Pictorial representation of the non-Hermitian quantum single-molecule junction (nHQSMJ) model. The leads are represented by two gray triangles. The spring portrays the molecule connecting the leads. The arrows with the cyan cusps depict the state of the two-level system (TLS), depending on its position along the junction. When the TLS is in the left lead, the arrow points downward. As the TLS moves toward the right lead, the arrow rotates until it reaches the upward position, which implies a complete transfer to the right lead. The sink below the right lead absorbs the TLS probability in an irreversible way. In the formalism, the sink is represented by a Hermitian decay operator. The decay operator acts on quantum states that in turn determine probabilities. Hence, the action of the sink unfolds upon an ensemble of trajectories of the TLS system. The picture also shows a box separating the molecular junction from the environment. The drawing of the box resembles on purpose that of an oven with a thermostat on top. The thermostat temperature is set at the same value of the $T_{R}$ temperature of the environment. The thermostat controls the temperature inside the oven so that it is equal to that of the environment. The yellow color inside the oven and around it, in the environment, conveys the idea of thermal equilibrium.

To the authors’ knowledge, the Hamiltonian model in Equation (36) provides a novel non-linear approach to the modeling of lossy molecular junctions in thermal baths. Figure 1 displays a pictorial representation of the non-Hermitian quantum single-molecule junction (nHQSMJ) model at constant temperature.

The abstract dynamics of the nHQSMJ model is obtained upon using the non-Hermitian Hamiltonian (36) in Equation (31). The abstract equations of motion can be represented using different basis sets. The approach described in [31] is based on the representation in the adiabatic basis. We also adopt such an approach here. The adiabatic Hamiltonian is defined as:

(38) $${\tilde{h}}_{\mathrm{ad}}^{m}\left(Q\right)={\hat{H}}^{m}+H_{B}^{m}\left(X\right)+{\tilde{H}}_{I}^{m}\left(Q\right)-\frac{P^{2}}{2}\;,$$

and the adiabatic basis is introduced through the eigenvalue equation:

(39) $${\tilde{h}}_{\mathrm{ad}}^{m}\left(Q\right)|\Phi_{\alpha }\left(Q\right)\rangle =E_{\alpha }|\Phi_{\alpha }\left(Q\right)\rangle \;.$$

In practice, the adiabatic states are a kind of dressed states of the quantum system when it interacts with approximately “frozen” classical degrees of freedom. The operator ${\hat{\Gamma }}^{m}$ is chosen in a phenomenological way. In the adiabatic basis, it is taken as:

(40) $${\hat{\Gamma }}^{m}=\frac{\gamma }{2}\left[\begin{array}{cc} 1 & 0\\ 0 & 0 \end{array}\right]\;.$$

In this way we describe a model where the excited state is in contact with a sink while the ground state is left unperturbed.

The abstract equation of motion for the non-normalized density matrix of the model is:

(41) $$\begin{array}{ccc} \frac{\partial }{\partial t}{\tilde{\Omega }}^{m}\left(t\right) & = & -i\left[{\tilde{H}}_{S}^{e},{\tilde{\Omega }}^{m}\left(t\right)\right]-{\left[\Gamma^{m},{\tilde{\Omega }}^{m}\left(t\right)\right]}_{+}+\frac{1}{2}{\left\{{\tilde{H}}_{S}^{m},{\tilde{\Omega }}^{m}\right\}}_{B^{m}}\\ & - & \frac{1}{2}{\left\{{\tilde{\Omega }}^{m},{\tilde{H}}_{S}^{m}\right\}}_{B^{m}}-\kappa_{S}^{m}{\tilde{\Omega }}^{m}\left(t\right)\\ & = & \left(-i{\tilde{L}}^{m}-i{\hat{L}}^{\Gamma^{m}}\right){\tilde{\Omega }}^{m}\left(t\right)\;. \end{array}$$

The antisymmetric matrix $B^{m}$ has the same structure of that in Equation (23), but it considers only the physical degrees of freedom of the harmonic modes. Accordingly, the phase space compressibility of the model becomes $\kappa_{S}^{m}=-\left({\dot{\Pi }}_{1}/\mu_{1}\right)-\left({\dot{\Pi }}_{2}/\mu_{2}\right)$.

Equation (41) introduces the two Liouville operators:

(42) $$\begin{array}{ccc} -i{\tilde{L}}^{m} & = & -i\left[{\tilde{H}}_{S}^{e},...\right]+\frac{1}{2}{\left\{{\tilde{H}}_{S}^{m},...\right\}}_{B^{m}}-\frac{1}{2}{\left\{...,{\tilde{H}}_{S}^{m}\right\}}_{B^{m}}-\kappa^{m}\left(...\right)\;, \end{array}$$

(43) $$\begin{array}{ccc} -i{\hat{L}}^{\Gamma^{m}} & = & -{\left[\Gamma^{m},...\right]}_{+}\;. \end{array}$$

The equation of motion (41) is represented into the adiabatic basis as:

(44) $$\begin{array}{ccc} \frac{d}{dt}{\tilde{\Omega }}_{\alpha \alpha^{\prime }}^{m}\left(t\right) & = & \sum_{\beta \beta^{\prime }}\left(-i{\tilde{L}}_{\alpha \alpha^{\prime },\beta \beta^{\prime }}^{m}-i{\hat{L}}_{\alpha \alpha^{\prime },\beta \beta^{\prime }}^{\Gamma^{m}}\right){\tilde{\Omega }}_{\beta \beta^{\prime }}^{m}\left(t\right)\;. \end{array}$$

In Equation (44) we introduced the two Liouville super-operators $-i{\tilde{L}}_{\alpha \alpha^{\prime },\beta \beta^{\prime }}^{m}$ and $-i{\hat{L}}_{\alpha \alpha^{\prime },\beta \beta^{\prime }}^{\Gamma^{m}}$. We first consider ${\tilde{L}}_{\alpha \alpha^{\prime },\beta \beta^{\prime }}^{m}$. We have:

(45) $$\begin{array}{ccc} -i{\tilde{L}}_{\alpha \alpha^{\prime },\beta \beta^{\prime }}^{m} & = & \left(-i\omega_{\alpha \alpha^{\prime }}-i{\tilde{L}}_{\alpha \alpha^{\prime }}^{m}-\kappa^{m}\right)\delta_{\alpha \beta }\delta_{\alpha^{\prime }\beta^{\prime }}+{\tilde{T}}_{\alpha \alpha^{\prime },\beta \beta^{\prime }}\;, \end{array}$$

where $\omega_{\alpha \alpha^{\prime }}=E_{\alpha }\left(Q\right)-E_{\alpha^{\prime }}\left(Q\right)$ is the Bohr frequency. The operator:

(46) $$\begin{array}{ccc} i{\tilde{L}}_{\alpha \alpha^{\prime }}^{m} & = & P\frac{\partial }{\partial Q}+\frac{1}{2}\left(F_{\alpha }\left(Q\right)+F_{\alpha^{\prime }}\left(Q\right)\right)\frac{\partial }{\partial P}+P\frac{\Pi_{1}}{\mu_{1}}\frac{\partial }{\partial P}+\\ & & -\frac{\Pi_{1}}{\mu_{1}}\frac{\partial }{\partial \Lambda_{1}}-\left(P^{2}-k_{B}T\right)\frac{\partial }{\partial \Pi_{1}}+\Pi_{1}\frac{\Pi_{2}}{\mu_{2}}\frac{\partial }{\partial \Pi_{1}}+\\ & & -\frac{\Pi_{2}}{\mu_{2}}\frac{\partial }{\partial \Lambda_{2}}-\left(\frac{\Pi_{1}^{2}}{\mu_{1}}-k_{B}T\right)\frac{\partial }{\partial \Pi_{2}} \end{array}$$

is a classical-like Liouville operator generating the dynamics of the physical coordinates *X* under the feedback of the fictitious coordinates Y. In Equation (46) we have also introduced the Hellmann–Feynman force:

(47) $$F_{\alpha }\left(Q\right)=-\frac{\partial E_{\alpha }\left(Q\right)}{\partial Q}\;.$$

This is the force acting on the harmonic oscillator when state α is occupied.

The adiabatic surfaces transition operator ${\tilde{T}}_{\alpha \alpha^{\prime },\beta \beta^{\prime }}$ is purely off-diagonal. In the adiabatic basis it is expressed as:

$$\begin{array}{ccc} T_{\alpha \alpha^{\prime },\beta \beta^{\prime }} & = & \delta_{\alpha^{\prime }\beta^{\prime }}P\cdot C_{\alpha \beta }\left(Q\right)\left(1+\frac{1}{2}{\tilde{S}}_{\alpha \beta }\cdot \frac{\partial }{\partial P}\right)\\ & + & \delta_{\alpha \beta }P\cdot C_{\alpha^{\prime }\beta^{\prime }}^{*}\left(Q\right)\left(1+\frac{1}{2}{\tilde{S}}_{\alpha^{\prime }\beta^{\prime }}^{*}\cdot \frac{\partial }{\partial P}\right)\;, \end{array}$$

where

(48) $$\begin{array}{ccc} C_{\alpha \beta } & = & \langle \Phi_{\alpha }\left(Q\right)|\frac{\partial }{\partial Q}|\Phi_{\beta }\left(Q\right)\rangle \;, \end{array}$$

(49) $$\begin{array}{ccc} {\tilde{S}}_{\alpha \beta } & = & \frac{\left(E_{\alpha }-E_{\beta }\right)}{P\cdot C_{\alpha \beta }\left(Q\right)}C_{\alpha \beta }\left(Q\right)\;. \end{array}$$

Equation (48) defines the adiabatic coupling vector $C_{\alpha \beta }$. This vector quantifies the superposition of the adiabatic states when *Q* changes. Equation (49) introduces the vector ${\tilde{S}}_{\alpha \beta }$. If dimensional coordinates were introduced, ${\tilde{S}}_{\alpha \beta }$ would have the dimension of momentum. Roughly speaking, ${\tilde{S}}_{\alpha \beta }$ provides the momentum variation when jumping from one adiabatic surface to the other.

We now consider ${\hat{L}}^{\Gamma^{m}}$. In general, a decay operator ${\tilde{\Gamma }}_{\alpha ,\beta }$ can always be decomposed in a diagonal part ${\tilde{\Gamma }}_{\alpha \beta }^{d}={\tilde{\Gamma }}_{\alpha \beta }\delta_{\alpha \beta }\;,$ and an off-diagonal part ${\tilde{\Gamma }}_{\alpha \beta }^{o}={\tilde{\Gamma }}_{\alpha \beta }\left(1-\delta_{\alpha \beta }\right)\;,$ so that ${\tilde{\Gamma }}_{\alpha ,\beta }\equiv {\tilde{\Gamma }}_{\alpha \beta }^{d}+{\tilde{\Gamma }}_{\alpha \beta }^{o}\;.$ However, in the case of the decay operator of the nHQSMJ model adopted here, and defined in Equation (40), we have ${\left({\tilde{\Gamma }}^{m}\right)}_{\alpha \beta }^{o}=0$. Hence, we have:

(50) $$iL_{\alpha \alpha^{\prime },\beta \beta^{\prime }}^{\Gamma^{m}}=\gamma_{\alpha \alpha^{\prime }}^{m}\delta_{\alpha \beta }\delta_{\alpha^{\prime }\beta^{\prime }}\;,$$

where $\gamma_{\alpha \alpha^{\prime }}^{m}=\Gamma_{\alpha \alpha }^{m}+\Gamma_{\alpha^{\prime }\alpha^{\prime }}^{m}$.

Using Equations (45) and (50), Equation (44) becomes:

(51) $$\begin{array}{ccc} \frac{d}{dt}{\tilde{\Omega }}_{\alpha \alpha^{\prime }}^{m}\left(t\right) & = & -\sum_{\beta \beta^{\prime }}\left[\left(i\omega_{\alpha \alpha^{\prime }}+\gamma_{\alpha \alpha^{\prime }}^{m}+i{\tilde{L}}_{\alpha \alpha^{\prime }}^{m}+\kappa_{S}^{m}\right)\delta_{\alpha \beta }\delta_{\alpha^{\prime }\beta^{\prime }}+{\tilde{T}}_{\alpha \alpha^{\prime },\beta \beta^{\prime }}\right]{\tilde{\Omega }}_{\beta \beta^{\prime }}^{m}\left(t\right)\;. \end{array}$$

Equation (51) provides the equation of motion for the density matrix of the nHQSMJ model at constant temperature. This equation can be implemented through a variety of algorithms [31,32,33,34].

## 6. Calculations and Results

We consider a situation where the molecule is weakly coupled to the leads. This implies that neglecting the action of the transition operator ${\tilde{T}}_{\alpha \alpha^{\prime },\beta \beta^{\prime }}$ in Equation (51), the phase-space-dependent density matrix is propagated by means of the sequential short-time propagation (SSTP) algorithm [31,32] as:

(52) $$\begin{array}{ccc} {\tilde{\Omega }}_{\alpha \alpha^{\prime }}^{m}\left(t\right) & = & \prod_{j=1}^{n_{\mathrm{step}}}\sum_{\alpha \alpha^{\prime }}e^{-i\left(\omega_{\alpha \alpha^{\prime }}-i\gamma_{\alpha \alpha^{\prime }}^{m}-i\kappa^{m}+{\tilde{L}}_{\alpha \alpha^{\prime }}^{m}\right)\tau }{\tilde{\Omega }}_{\alpha \alpha^{\prime }}^{m},\left(0\right)\\ & = & \prod_{j=1}^{n_{\mathrm{step}}}\sum_{\alpha \alpha^{\prime }}e^{-i{\tilde{L}}_{\alpha \alpha^{\prime }}^{m,(0)}\tau }{\tilde{\Omega }}_{\alpha \alpha^{\prime }}^{m}\left(0\right)\;, \end{array}$$

where $\tau =t/n_{\mathrm{step}}$ and the non-Hermitian adiabatic Liouville super-operator is $-i{\tilde{L}}_{\alpha \alpha^{\prime }}^{m(0)}=-i\omega_{\alpha \alpha^{\prime }}-\gamma_{\alpha \alpha^{\prime }}^{m}-\kappa_{S}^{m}-i{\tilde{L}}_{\alpha \alpha^{\prime }}^{m}$. Quantum statistical averages can be calculated as:

(53) $$\begin{array}{ccc} \tilde{T}r^{m}\left({\tilde{\rho }}^{m}\left(t\right){\tilde{\chi }}^{m}\right) & = & \frac{\sum_{\alpha \alpha^{\prime }}\int dX^{m}{\tilde{\Omega }}_{\alpha \alpha^{\prime }}^{m}\left(X^{m},t\right){\tilde{\chi }}_{\alpha^{\prime }\alpha }\left(X^{m}\right)}{\sum_{\sigma }\int dX^{m}{\tilde{\Omega }}_{\sigma \sigma }^{m}\left(X^{m},t\right)}\\ & = & \frac{\sum_{\alpha \alpha^{\prime }}{\langle {\tilde{\Omega }}_{\alpha \alpha^{\prime }}^{m}\left(t\right){\tilde{\chi }}_{\alpha^{\prime }\alpha }\rangle }^{m}}{\sum_{\sigma }{\langle {\tilde{\Omega }}_{\sigma \sigma }^{m}\left(t\right)\rangle }^{m}}\;, \end{array}$$

where the bracket ${\langle \ldots \rangle }^{m}$ stands for the average in the extended phase space of the model. We implement Equation (53) in the following way [31,32]. Once the quantum initial state is assigned for every point of the extended phase space, the calculation of quantum averages can be performed by sampling the initial $X^{e}$ with a Monte Carlo algorithm. The point $X^{m}$ is then propagated over the assigned quantum state for a time length *t*. The time step $\tau =t/n_{\mathrm{step}}$ in Equation (53) must be chosen and small enough to minimize the numerical error. In the calculation we take the following uncorrelated form of the initial phase-space-dependent density matrix:

(54) $${\tilde{\Omega }}^{e}\left(X^{m},0\right)={\hat{\Omega }}^{m}\left(0\right)\Omega_{B}^{m}\left(X,0\right)\Omega_{\mathrm{nhc}}^{m}\left(Y,0\right)\;,$$

where

(55) $$\begin{array}{ccc} {\hat{\Omega }}^{m}\left(0\right) & = & \left[\begin{array}{cc} 0 & 0\\ 0 & 1 \end{array}\right]\;, \end{array}$$

(56) $$\begin{array}{ccc} \Omega_{B}^{m}\left(X,0\right) & = & \frac{\tanh(\beta \omega /2)}{\pi }\exp\left(-\frac{\tanh(\beta \omega /2)}{\omega }H_{B}^{m}\left(X\right)\right)\;, \end{array}$$

(57) $$\begin{array}{ccc} \Omega_{\mathrm{nhc}}^{m}\left(Y,0\right) & = & \frac{\prod_{J=1}^{2}\delta \left(\Lambda_{J}\right)\exp\left(-\Pi_{J}^{2}/2\right)}{Z_{Y}^{m}} \end{array}$$

and

(58) $$\int dY\prod_{J=1}^{2}\delta \left(\Lambda_{J}\right)\exp\left(-\Pi_{J}^{2}/2\sigma^{2}\right).$$

Calculations were performed with fixed values of γ=0.1, ω=1/3, Δ=1, and c=0.007, time step τ=0.005, number of time steps $n_{\mathrm{step}}=10^{4}$, and number of Monte Carlo steps $n_{\mathrm{mcs}}=25\cdot 10^{3}$. All the values of the parameters are given in a dimensional units. A total of 20 different values of the inverse temperature were considered upon chosing $\beta_{l+1}=1/T_{l+1}=\beta_{0}\left(1+l\right)$, with l=0,…,19 and $\beta_{0}=0.0005$. For each $\beta_{l}$ we have investigated four different types of dynamics: (i) Unitary dynamics, (ii) constant-temperature dynamics, (iii) non-unitary dynamics, and (iv) non-unitary dynamics at constant temperature. Initial conditions are chosen with the two-level system in its ground state and the bath in a thermal state. It is useful to introduce ${\hat{\Xi }}^{m}$ and ${\hat{X}}^{m}$, the non-normalized and normalized reduced density matrices, respectively. We write their matrix elements in the adiabatic basis as:

(59) $$\begin{array}{ccc} \Xi_{\alpha \alpha^{\prime }}^{m} & = & \int dX^{m}{\tilde{\Omega }}_{\alpha \alpha^{\prime }}\;, \end{array}$$

(60) $$\begin{array}{ccc} X_{\alpha \alpha^{\prime }}^{m} & = & \int dX^{m}{\tilde{\rho }}_{\alpha \alpha^{\prime }}\;. \end{array}$$

Both reduced density matrices can be easily calculated by means of our numerical approach.

**Figure 2:**
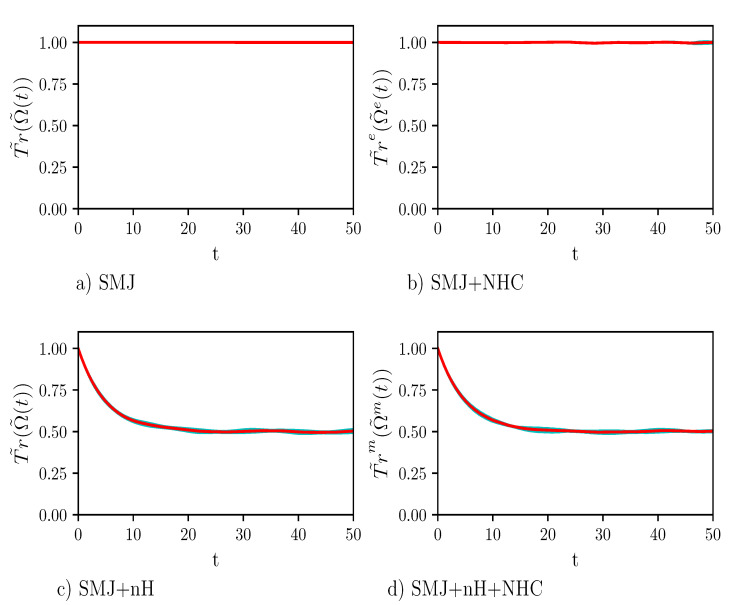
Plot of $\tilde{T}r^{}\left({\tilde{\Omega }}^{}\left(t\right)\right)$. The values of the parameters of the calculation are: number of time steps = 10,000, number of Monte Carlo points =2500, time step τ=0.005, γ=0.1, ω=1/3, Δ=1, coupling constant c=0.007, and inverse temperature β=0.0050. The red line shows the average trend while the cyan area around it, which is hardly visible, displays the statistical error of the calculation. Panels (**a**) and (**b**) show the results of the calculations when the dynamics takes place for the isolated single-molecule junction (SMJ) dynamics and for the SMJ dynamics at constant temperature, i.e., SMJ+NHC dynamics, respectively. Panel (**c**) shows the result of the calculation when a decay operator acts onto the SMJ, which is for SMJ+nH dynamics. Finally, in Panel (**d**) all types of dynamics are considered together, i.e., SMJ+nH+NHC dynamics. It is easy to see that the trace is conserved when the Hamiltonian is Hermitian. Instead, the population drops to about one half in the case of nH dynamics.

In order to check the numerical scheme, we calculated the evolution in time of $\tilde{T}r\left({\tilde{\Omega }}^{m}\left(t\right)\right)$. The results are shown in Figure 2. When the decay operator is set to zero, Panels (a) and (b), the trace of the density matrix is conserved with extremely good numerical precision both when the dynamics is unitary and when the temperature is controlled. This provides a convincing indication that our algorithm conserves important dynamical invariants. Panels (c) and (d) in Figure 2 show the decrease in time of $\tilde{T}r\left({\tilde{\Omega }}^{m}\left(t\right)\right)$ non-unitary dynamics. Moreover, there is no appreciable difference between unitary and constant-temperature dynamics.

**Figure 3:**
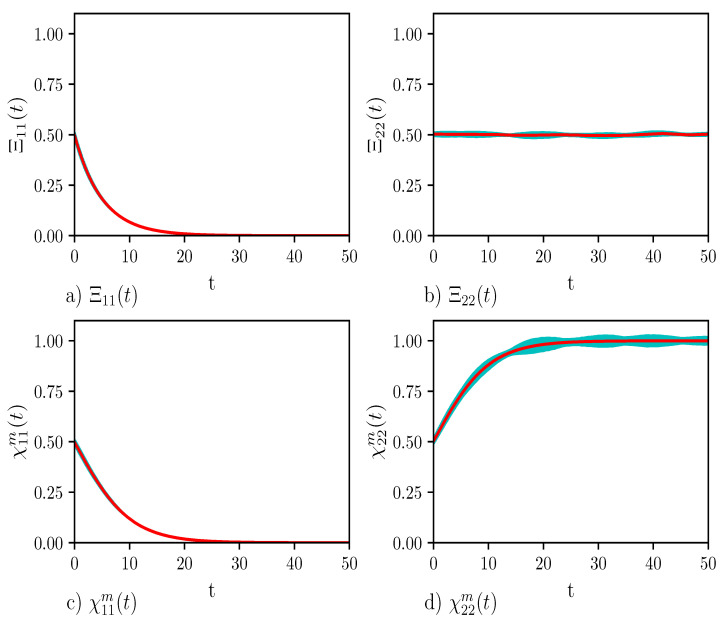
The plots show the diagonal elements of the density matrices resulting from nH+NHC dynamics. Panels (**a**) and (**b**) show the behavior of the non-normalized density matrix elements $\Xi_{11}\left(t\right)$ and $\Xi_{22}\left(t\right)$, respectively. Panels (**c**) and (**d**) display the behavior of the normalized density matrix elements $X_{11}^{m}\left(t\right)$ and $X_{22}^{m}\left(t\right)$. They can be discussed by looking at Panel (**d**) of Figure 2. The values of the parameters of the calculations are: number of time steps =10,000, number of Monte Carlo points =2500, time step τ=0.005, γ=0.1, ω=1/3, Δ=1, c=0.007, and β=0.0050. The red line is the average trend, the cyan area (almost invisible to the eye) is the error. In Panel (**a**) the effects of the decay operator ${\hat{\Gamma }}^{m}$ can be observed. The trend in Panel (**b**) clarifies the cause for the evolution of the trace in Figure 2. There is no difference between Panel (**a**,**c**) since the trace decreases because of the decrease of $\Omega_{11}$. The comparison between Panel (**b**,**d**) and further clarifies that the cause of the population loss of the excited state is the action of the decay operator and not the energy transfer.

In Figure 3 we show the time-evolution of $\Xi_{\alpha \alpha }^{m}\left(t\right)$ and $X_{\alpha \alpha }^{m}{\left(t\right)\rangle }^{m}$, with α=1,2, defined in Equations (59) and (60), respectively. Panels (a) and (b) display $\Xi_{11}^{m}\left(t\right)$ and $\Xi_{22}^{m}\left(t\right)$, respectively. Initially, adiabatic states are equally occupied. However, according to Equation (40), only the occupation of the excited state experiences depletion, see in Panel (a). Panels (c) and (d) of Figure 3 show $X_{11}^{m}\left(t\right)$ and $X_{22}^{m}\left(t\right)$, respectively. The behavior of $X_{11}^{m}\left(t\right)$ is hardly distinguishable from that of $\Xi_{11}^{m}\left(t\right)$. However, the situation is different for $X_{22}^{m}\left(t\right)$ in Panel (d). As a matter of fact, while $\Xi_{22}^{m}\left(t\right)$ is constant, $X_{22}^{m}\left(t\right)$ increases so that $\tilde{T}r^{m}\left({\tilde{\rho }}^{m}\left(t\right)\right)=X_{11}^{m}\left(t\right)+X_{22}^{m}\left(t\right)=1$.

**Figure 4:**
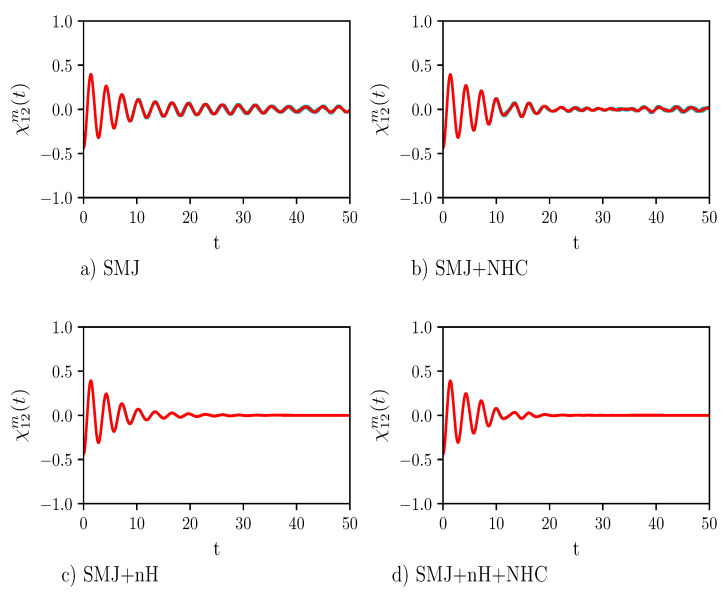
The plot shows the real part of $X_{12}^{m}\left(t\right)$, which is the off-diagonal element of the reduced normalized density matrix. Panels (**a**) and (**b**) show the results of the calculations when the dynamics takes place for the isolated single-molecule junction (SMJ) dynamics and for the SMJ dynamics at constant temperature, i.e., SMJ+NHC dynamics, respectively. Panel (**c**) shows the result of the calculation when a decay operator acts onto the SMJ, SMJ+nH. Finally, in Panel (**d**) all types of dynamics are considered together, i.e., SMJ+nH+NHC dynamics. The values of the parameters of the calculations are: mumber of time steps =10,000, number of Monte Carlo points =2500, time step τ=0.005, γ=0.1, ω=1/3, Δ=1, c=0.007, and β=0.0050. The red line is the average trend, the cyan area (which is almost invisible to the eye) is the error. The trend clearly shows decoherence.

In Figure 4 we show the real part of the reduced off-diagonal element Re(X12m(t)). For the chosen values of the parameters, dephasing occurs on the same time scale of the population’s depletion of the excited state.

**Figure 5:**
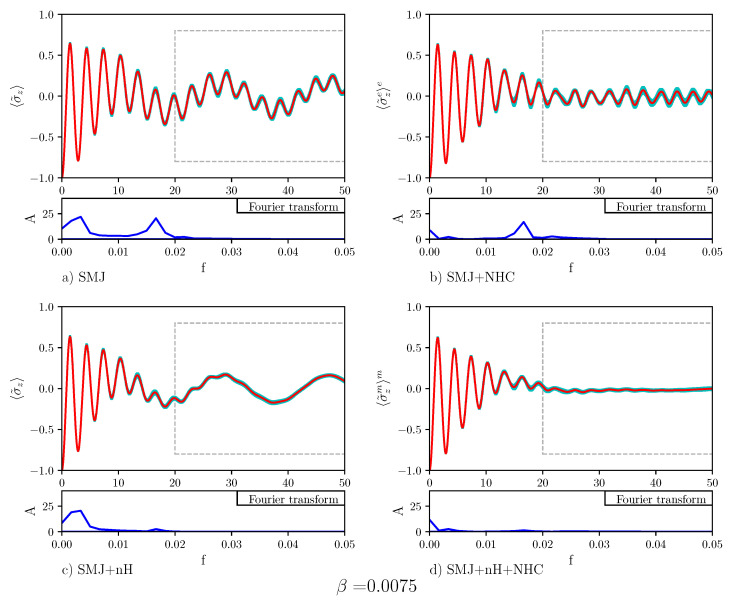
The plot shows the average population transfer vs. time for different types of dynamics. The values of the parameters of the calculation are number of time steps = 10,000, number of Monte Carlo points = 2500, τ=0.005, γ=0.1, ω=1/3, Δ=1, g=0.007, and β=0.0075). The red line indicates the average trend, the cyan area represents the error. The blue line denotes the Fourier transform of the time evolution of the average value (the data are considered after t=20). Panel (**a**) displays the results for the SMJ dynamics. One can observe that the values oscillate with low and high frequency. Panel (**b**) displays the dynamics of the SMJ with temperature control (SMJ+NHC dynamics). The thermostat facilitates the population transfer and stabilizes its average. The low frequency terms are highly damped while the high frequency ones are unaltered. Panel (**c**) shows that the effect of the decay operator (SMJ+nH dynamics) is to cancel the high frequency term. Finally, in (**d**), it is shown that the union of the NHC thermostat and decay operator (SMJ+nH+NHC dynamics) determines a stable tranfer. Both the low and the high frequency terms are strongly damped.

**Figure 6:**
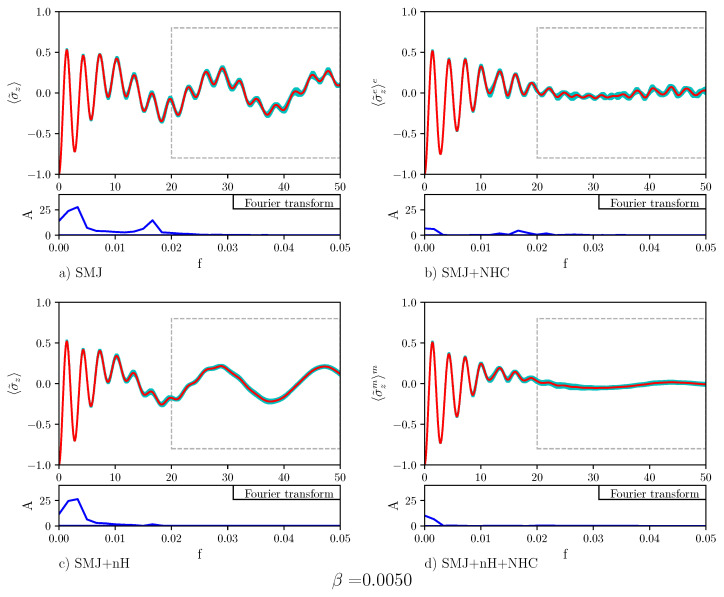
The plot shows the average population difference vs. time for different types of dynamics. The values of the calculation parameters are: number of time steps = 10,000, number of Monte Carlo points = 2500, τ=0.005, γ=0.1, ω = 1/3, Δ = 1, g = 0.007, and β = 0.0050). The red line indicates the average trend, the cyan area represents the error. The blue line denotes the Fourier transform of the time evolution of the average value (the data are considered after t=20). Panel (**a**) displays an average population transfer for the SMJ dynamics where low frequency oscillations are strongly damped. In Panel (**b**) it is shown that a higher temperature damps high frequency oscillations in SMJ+NHC dynamics. Statistical error appears to be somewhat greater. Panel (**c**) shows that the effect of the decay operator is to damp high frequency oscillations in SMJ+nH dynamics. Panel (**d**) shows that the combined action of the NHC thermostat and the decay operator, i.e., SMJ+nH+NMJ dynamics determines a stable transfer process. Both low and high frequency oscillations are strongly damped, except for a very weak oscillation at a very low frequency.

**Figure 7:**
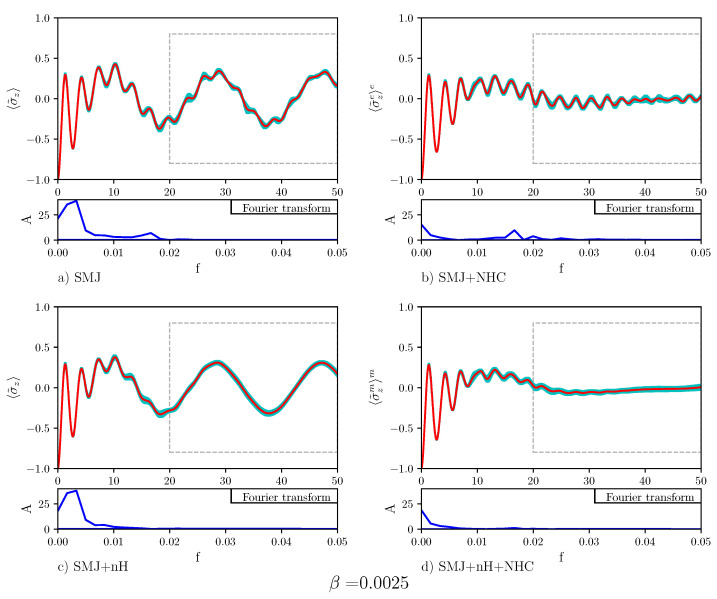
The plot shows the average population transfer vs. time for different types of dynamics. NMDS = 10,000, NMCP = 2500, τ=0.005, γ=0.1, ω=1/3, Δ=1, g=0.007, and β=0.0025. The red line is the average trend, the cyan area is the error. The blue line is the Fourier transform, carried out after the stabilization of the trend (from t=20 onward). In Panel (**a**) the population difference oscillates with low and high frequency contributions (for SMJ dynamics). Panel (**b**) shows that the thermostat (SMJ+NHC dynamics) facilitates the population transfer and stabilizes it more than in the case of lower temperatures. The low frequency terms are highly damped while the high frequency ones are unaltered. Panel (**c**) shows that the effect of the decay operator (case of the SMJ+nH dynamics) is to cancel the high frequency term. Finally, Panel (**d**) shows that the SMJ+nH+NHC dynamics displays a stable transfer: Both the low and the high frequency terms are strongly damped.

Figure 5 displays the time evolution of the population difference for β=0.0075. Below each plot showing the time evolution of the population difference, the Fourier transform is also displayed. The Fourier transform of Panel (a) displays two peaks: One at a small frequency and one at a higher frequency. The Rabi-like oscillations of the population difference at a distant time are signs of the absence of stable quantum transport in the nHQSMJ model. Nosé-Hoover chain dynamics, shown in Panel (b), suppresses the peak at small frequency. The Rabi-like oscillations of the population difference at distant time are less pronounced: They take place at a relatively high frequency, as the Fourier transform shows, around zero. This indicates that in the presence of thermal noise the population difference oscillates around zero so that transport is stable. Figure 6 shows the time evolution of the population difference β=0.0050, which corresponds to a temperature higher than that in Figure 5. All the effects shown by Figure 5 appear more marked in Figure 6. This can be seen as a particular instance of environment-assisted quantum transport: Remarkably, the noise provided by an environment can defeat Anderson localization [41] and assist quantum transport. The idea that the noise of the environment could enhance quantum transport, instead of suppressing it, has been postulated to explain the high efficiency of energy transport in photosynthetic systems [42]. Obviously, the noise-enhancement of quantum transport can take place only below a certain threshold. Above it, transport is suppressed by the quantum Zeno effect [43]. In Panel (c), Hamiltonian dynamics of the bath is combined with a non-zero decay operator acting on the two-level system. As seen, the decay operator suppresses the high frequencies in the Rabi-like oscillations of the population difference at distant time. Such oscillations show that, even in this case, quantum transport is not very stable. Comparison of the dynamics in Panel (b) and in Panel (c) shows that there is a difference between thermal and probability sink dissipation: The first suppresses low frequency oscillation while the second damps high frequency oscillations. As one can expect, the combination of the two dynamics, shown in Panel (d), leads to the suppression of both frequencies, increasing the efficiency and the stability of the population transport. Calculations for β=0.0025 are reported in Figure 7. The results are indistinguishable to the human eye from those obtained at β=0.0075. This reinforces the conclusion that quantum transport in the nHQSMJ model is enhanced by coupling the two-level system with a probability sink and controlling the temperature of the molecule.

## 7. Conclusions

In this work, we constructed a theory for studying non-Hermitian phase-space-dependent quantum systems at constant temperature. This theory is based on an operator-valued Wigner formulation of quantum mechanics or, in other words, on a phase-space-dependent density matrix. The condition of constant temperature in phase space was achieved by means of the Nosé-Hoover chain thermostat. A mathematical result of the formalism is the derivation of the non-linear equation of motion for the normalized phase-space-dependent density matrix of the system. We remark that this result, considering a constant temperature bath, improved the theory developed in [15] where the temperature fluctuations were not constrained. These latter situation is somewhat unrealistic in common experiments.

This theory was applied to a model of a non-Hermitian quantum single-molecule junction. We emphasize that this model treated probability loss and thermal fluctuations on the same level. In detail, the model comprised of a two-level system with a probability sink coupled to a thermalized harmonic mode. The structure of our model was conjectured upon drawing an analogy with the process of noise-assisted transport [44,45,46]. In our case, we expect that the assisted quantum transport arises from the combined action of the probability sink and the constant-temperature fluctuating molecule. Indeed, we observed a transport enhancement in silico upon simulating numerically the non-Hermitian quantum single-molecule junction model for a range of temperatures, while keeping the values of the other parameters constant. The Fourier transformed signal in frequency space showed that the Nosé-Hoover chain thermostat suppresses the slow frequencies while the probability sink damps the high frequency oscillations of the population difference of the non-Hermitian quantum single-molecule junction model at a constant temperature.

The non-Hermitian quantum single-molecule junction model at constant temperature introduced in this paper could more accurately be classified as a particular instance of a class of models. As a matter of fact, it could be easily generalized by considering a greater number of quantum states (∼10) and an even greater number of classical modes ($∼10^{3}-10^{5}$). Of course, more sophisticated algorithms would have to be used for calculating the evolution of the density matrix. We also note that spin models that were similar but simpler than the non-Hermitian quantum single-molecule junction model introduced in this paper could be solved analytically when obeying certain symmetry properties [47,48,49,50,51,52,53,54,55]. It would be interesting to investigate whether the extension of these models to non-Hermitian quantum mechanics would still be analytically treatable. We defer such generalizations to future work.

## Data Availability

Data available on request.

## Abbreviations

The following abbreviations are used in this manuscript:

nHQSMJ	non-Hermitian quantum single-molecule junction
NHQM	non-Hermitian quantum mechanics
TLS	two-level system
SMJ	single-molecule junction
NHC	Nosé-Hoover chain
nH	non-Hermitian

## Appendix A. Time Evolution of the Trace of $\tilde{\Omega }\left(t\right)$

The trace of Equation (11):

(A1) $$\begin{array}{ccc} \frac{d}{dt}\tilde{T}r\left(\tilde{\Omega }\left(t\right)\right) & = & -2\tilde{T}r\left(\hat{\Gamma }\tilde{\Omega }\left(X,t\right)\right)+\frac{1}{2}\tilde{T}r\left({\left\{{\tilde{H}}^{e},\tilde{\Omega }\left(t\right)\right\}}_{B}\right)\\ & - & \frac{1}{2}\tilde{T}r\left({\left\{\tilde{\Omega }\left(t\right),\tilde{H}\right\}}_{B}\right)\;. \end{array}$$

The last term in the right hand side of Equation (A1) can be transformed as follows:

(A2) $$\begin{array}{ccc} -\frac{1}{2}\tilde{T}r\left({\left\{\tilde{\Omega }\left(t\right),\tilde{H}\right\}}_{B}\right) & = & -\frac{1}{2}\tilde{T}r\left(\sum_{I,J=1}^{2N}\frac{\partial \tilde{\Omega }\left(t\right)}{\partial X_{I}}B_{IJ}\frac{\partial \tilde{H}}{\partial X_{J}}\right)=-\frac{1}{2}\tilde{T}r\left(\sum_{I,J=1}^{2N}\frac{\partial \tilde{\Omega }\left(t\right)}{\partial X_{J}}B_{JI}\frac{\partial \tilde{H}}{\partial X_{I}}\right)\\ & = & \frac{1}{2}\tilde{T}r\left(\sum_{I,J=1}^{2N}\frac{\partial \tilde{H}}{\partial X_{I}}B_{IJ}\frac{\partial \tilde{\Omega }\left(t\right)}{\partial X_{J}}\right)=\frac{1}{2}\tilde{T}r\left({\left\{\tilde{H},\tilde{\Omega }\left(t\right)\right\}}_{B}\right) \end{array}$$

Substituting Equation (A2) in the right hand side of Equation (A1), one obtains:

(A3) $$\begin{array}{ccc} \frac{d}{dt}\tilde{T}r\left(\tilde{\Omega }\left(t\right)\right) & = & -2\tilde{T}r\left(\hat{\Gamma }\tilde{\Omega }\left(X,t\right)\right)+\tilde{T}r\left({\left\{\tilde{H},\tilde{\Omega }\left(t\right)\right\}}_{B}\right)\;. \end{array}$$

However, the last term in the right hand side of Equation (A3) is zero:

(A4) $$\begin{array}{ccc} \tilde{T}r\left({\left\{\tilde{H},\tilde{\Omega }\left(t\right)\right\}}_{B}\right) & = & \tilde{T}r\left(\sum_{I,J=1}^{2N}\frac{\partial \tilde{H}}{\partial X_{I}}B_{IJ}\frac{\partial \tilde{\Omega }\left(t\right)}{\partial X_{J}}\right)=-\tilde{T}r\left(\sum_{I,J=1}^{2N}\frac{\partial^{2}\tilde{H}}{\partial X_{I}\partial X_{J}}B_{IJ}\tilde{\Omega }\left(t\right)\right)\\ & = & 0 \end{array}$$

because of the antisymmetry of B. Equation (A4) allows one to obtain Equation (15).

## Appendix B. Time Evolution of the Trace of ${\tilde{\Omega }}^{e}\left(t\right)$.

The trace of Equation (26) is:

(A5) $$\frac{d}{dt}\tilde{T}r^{e}\left({\tilde{\Omega }}^{e}\left(t\right)\right)=-2\tilde{T}r^{e}\left(\hat{\Gamma }{\tilde{\Omega }}^{e}\left(t\right)\right)+\frac{1}{2}\tilde{T}r^{e}\left({\left\{{\tilde{H}}^{e},{\tilde{\Omega }}^{e}\right\}}_{B^{e}}\right)-\frac{1}{2}\tilde{T}r^{e}\left({\left\{{\tilde{\Omega }}^{e},{\tilde{H}}^{e}\right\}}_{B^{e}}\right)-\tilde{T}r^{e}\left(\kappa^{e}{\tilde{\Omega }}^{e}\left(t\right)\right)\;.$$

One can find the following identity:

(A6) $$\begin{array}{ccc} \tilde{T}r^{e}\left({\left\{{\tilde{\Omega }}^{e},{\tilde{H}}^{e}\right\}}_{B^{e}}\right) & = & \tilde{T}r^{e}\left(\sum_{I,J=1}^{2(N+2)}\frac{\partial {\tilde{\Omega }}^{e}}{\partial X_{I}^{e}}B_{IJ}^{e}\frac{\partial {\tilde{H}}^{e}}{\partial X_{J}^{e}}\right)=\tilde{T}r^{e}\left(\sum_{I,J=1}^{2(N+2)}\frac{\partial {\tilde{\Omega }}^{e}}{\partial X_{J}^{e}}B_{JI}^{e}\frac{\partial {\tilde{H}}^{e}}{\partial X_{I}^{e}}\right)\\ & = & \tilde{T}r^{e}\left(\sum_{I,J=1}^{2(N+2)}\frac{\partial {\tilde{H}}^{e}}{\partial X_{I}^{e}}B_{JI}^{e}\frac{\partial {\tilde{\Omega }}^{e}}{\partial X_{J}^{e}}\right)=-\tilde{T}r^{e}\left(\sum_{I,J=1}^{2(N+2)}\frac{\partial {\tilde{H}}^{e}}{\partial X_{I}^{e}}B_{IJ}^{e}\frac{\partial {\tilde{\Omega }}^{e}}{\partial X_{J}^{e}}\right)\\ & = & -\tilde{T}r^{e}\left({\left\{{\tilde{H}}^{e},{\tilde{\Omega }}^{e}\right\}}_{B^{e}}\right) \end{array}$$

Inserting Equation (A6) in Equation (A5) one obtains:

(A7) $$\frac{d}{dt}\tilde{T}r^{e}\left({\tilde{\Omega }}^{e}\left(t\right)\right)=-2\tilde{T}r^{e}\left(\hat{\Gamma }{\tilde{\Omega }}^{e}\left(t\right)\right)+\tilde{T}r^{e}\left({\left\{{\tilde{H}}^{e},{\tilde{\Omega }}^{e}\right\}}_{B^{e}}\right)-\tilde{T}r^{e}\left(\kappa^{e}{\tilde{\Omega }}^{e}\left(t\right)\right)\;.$$

One can now find the following identity:

(A8) $$\begin{array}{ccc} \tilde{T}r^{e}\left({\left\{{\tilde{H}}^{e},{\tilde{\Omega }}^{e}\right\}}_{B^{e}}\right) & = & -\tilde{T}r^{e}\left({\left\{{\tilde{H}}^{e},{\tilde{\Omega }}^{e}\right\}}_{B^{e}}\right)=-\tilde{T}r^{e}\left(\sum_{I,J=1}^{2(N+1)}\frac{\partial {\tilde{H}}^{e}}{\partial X_{I}^{e}}B_{IJ}^{e}\frac{\partial {\tilde{\Omega }}^{e}}{\partial X_{J}^{e}}\right)\\ & = & \tilde{T}r^{e}\left(\sum_{I,J=1}^{2(N+1)}\frac{\partial B_{IJ}^{e}}{\partial X_{J}^{e}}\frac{\partial {\tilde{H}}^{e}}{\partial X_{I}^{e}}{\tilde{\Omega }}^{e}\right)=\tilde{T}r^{e}\left(\kappa^{e}{\tilde{\Omega }}^{e}\right)\;. \end{array}$$

Equation (A8) is obtained exploiting the identity:

(A9) $$\sum_{I,J=1}^{2(N+1)}B_{IJ}^{e}\frac{\partial^{2}{\tilde{H}}^{e}}{\partial X_{I}^{e}\partial X_{J}^{e}}\equiv 0\;,$$

which derives from the fact that the trace of a symmetric matrix times an antisymmetric matrix is identically zero. Finally, substituting Equation (A8) in Equation (A7), one obtains Equation (28).
